# Designing a Next-Generation Multiepitope-Based Vaccine against *Staphylococcus aureus* Using Reverse Vaccinology Approaches

**DOI:** 10.3390/pathogens12030376

**Published:** 2023-02-25

**Authors:** Soumya Ranjan Mahapatra, Jyotirmayee Dey, T. Kiran Raj, Namrata Misra, Mrutyunjay Suar

**Affiliations:** 1 School of Biotechnology, Kalinga Institute of Industrial Technology (KIIT), Deemed to be University, Bhubaneswar 751024, India; 2 Department of Biotechnology and Bioinformatics, School of Life Sciences, JSS Academy of Higher Education & Research, Mysuru 570015, India; 3 KIIT-Technology Business Incubator (KIIT-TBI), Kalinga Institute of Industrial Technology (KIIT), Deemed to be University, Bhubaneswar 751024, India

**Keywords:** *Staphylococcus aureus*, CnBP, epitopes, vaccine, immunoinformatic

## Abstract

*Staphylococcus aureus* is a human bacterial pathogen that can cause a wide range of symptoms. As virulent and multi-drug-resistant strains of *S. aureus* have evolved, invasive *S. aureus* infections in hospitals and the community have become one of the leading causes of mortality and morbidity. The development of novel techniques is therefore necessary to overcome this bacterial infection. Vaccines are an appropriate alternative in this context to control infections. In this study, the collagen-binding protein (CnBP) from *S. aureus* was chosen as the target antigen, and a series of computational methods were used to find epitopes that may be used in vaccine development in a systematic way. The epitopes were passed through a filtering pipeline that included antigenicity, toxicity, allergenicity, and cytokine inducibility testing, with the objective of identifying epitopes capable of eliciting both T and B cell-mediated immune responses. To improve vaccine immunogenicity, the final epitopes and phenol-soluble modulin α4 adjuvant were fused together using appropriate linkers; as a consequence, a multiepitope vaccine was developed. The chosen T cell epitope ensemble is expected to cover 99.14% of the global human population. Furthermore, docking and dynamics simulations were used to examine the vaccine’s interaction with the Toll-like receptor 2 (TLR2), revealing great affinity, consistency, and stability between the two. Overall, the data indicate that the vaccine candidate may be extremely successful, and it will need to be evaluated in experimental systems to confirm its efficiency.

## 1. Introduction

The “ESKAPE” pathogens are common causes of potentially lethal nosocomial infections in severely ill and immunocompromised patients, and they are distinguished by potential drug resistance mechanisms [1]. One of the ESKAPE bacteria, *Staphylococcus aureus*, is a potential pathogen that can cause a variety of nosocomial infections in humans, ranging from mild skin infections to systemic infections, having an annual incidence rate ranging from 20 to 50 cases per 100,000 people [2]. Because of its resistance to multiple antibiotics, toxin-mediated virulence, and invasiveness, *S. aureus* was recognized as one of the most serious threats to human health by the World Health Organization in 2017. Antibiotic resistance further complicates the treatment of *S. aureus* infections [3], resulting in increased morbidity, mortality, and rising healthcare costs [4].

*S. aureus* is a highly versatile gram-positive, catalase-positive, coagulase-positive, non-motile coccus bacterium that colonizes skin and nasopharynx. *S. aureus* has an extraordinary repertoire of virulence factors, including microbial surface components recognizing adhesive matrix molecules (MSCRAMMs), which allows it to survive in extreme conditions and help in binding to extracellular matrix proteins in the human host [5,6]. The collagen (Cn)-binding protein (CnBP) is an MSCRAMM prototype as an adhesion and immune evasion factor. It plays a significant role in staphylococcal pathogenesis. The *S. aureus* surface protein CnBP has been demonstrated to be an effective target for antibody-mediated methods and virulence factors, with the strength of collagen adherence corresponding with disease severity [7,8,9,10]. In addition, CnBP binds to complement protein C1q and prevents the classical pathway of complement fixation. Because of the enormous rise in nosocomial staphylococcal infections, especially multidrug-resistant *S. aureus*, new treatment options, including vaccines and therapeutic antibodies, are urgently needed. The development of safe and efficient bacterial vaccines that target specific antigens or toxins, notably capsular polysaccharides, has been achieved before. The tetanus toxoid and pneumococcal conjugate vaccines are two of the most well-known examples [11,12]. Despite the fact that a number of vaccine candidates have shown promise in preclinical testing in a variety of animal models, those that have advanced to late-stage clinical testing have failed to show effectiveness in human trials [13]. The main challenge in developing an effective *S. aureus* vaccine is that this organism can produce a wide range of possible virulence factors. This obstacle may be overcome by using epitopes that trigger a humoral as well as a cellular immunological response.

Subunit vaccines, which are mostly composed of T and B cell epitopes, have recently received a lot of attention in the field of vaccinology [14,15]. T cells are important for the generation of opsonizing antibodies and promote phagocytosis by recruiting neutrophils and macrophages from the bone marrow to the site of Staphylococcus aureus infection [16]. It may be possible to induce T cells that are capable of increasing phagocyte recruitment to sites of infection and facilitate the clearance of S. aureus from tissues [17]. B cells are crucial for the progression and also have important antigen-presenting cells, and some of them were proven to be important for antigen uptake and thereby enhanced macrophage activity in S. aureus [18]. When compared to killed and attenuated vaccines, epitope-based vaccines have less adverse effects, no danger of pathogenicity, since they do not replicate in the host, and are more cost effective to produce [19]. A good vaccine candidate, on the other hand, must be highly antigenic, conserved, and non-pathogenic [20]. As a result, CnBP is being considered as a potential target for the discovery of novel epitopes as vaccine candidates capable of eliciting an immune response that is both humoral and cellular in nature against *S. aureus*. Despite the fact that recent immunoinformatic studies using various tools and databases have revealed a few possible T cell and B cell epitopes from *S. aureus* antigens [21,22,23,24,25], no efforts have been made to identify epitopes using the CnBP protein, which has been identified as a potential vaccine target [26].

**Figure 1:**
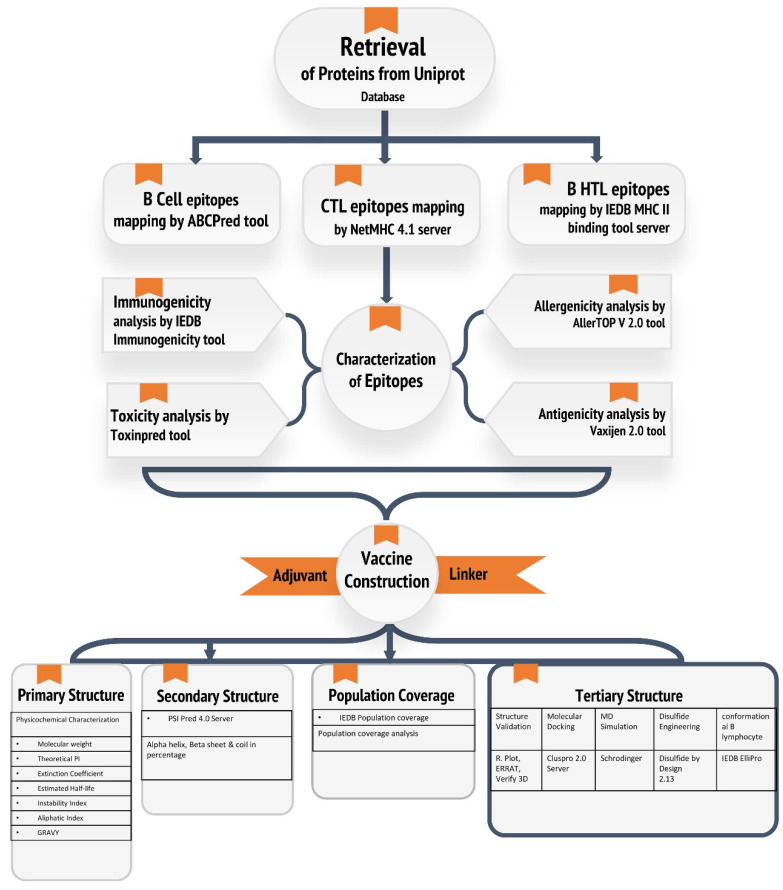
A schematic illustration of the immunoinformatics-guided design of a multi-epitope vaccine against *S. aureus*.

In this context, bioinformatic-based approaches can make a contribution to the design of peptide-based vaccines. As a result, the goal of this research was to detect B and T epitopes within the amino acid sequence of CnBP in order to develop a subunit vaccine against *S. aureus* infections in silico. Furthermore, to improve vaccine efficiency, the final multi-epitope vaccine design was constructed by integrating the best epitopes, phenol-soluble modulin 4 as an adjuvant, and appropriate linkers. Following that, the vaccine’s physicochemical characteristics, as well as secondary and tertiary structures, were anticipated. In addition, docking and dynamics simulations were run to reveal vaccine construct binding stability with the TLR2 receptor. Finally, in silico cloning was employed to confirm the vaccine construct’s expression. The findings of this study could aid in the development of highly effective vaccines, so as to curtail the threat of infections caused by this pathogen. Figure 1 shows the overall computational workflow used in this study.

## 2. Materials and Methods

### 2.1. Retrieval of Protein Sequence

CnBP was chosen as the antigenic protein for this study (accession no.: Q53654). For further analysis, the complete protein sequence was extracted from UniProtKB (https://www.uniprot.org/, accessed on 10 February 2023) in the FASTA format.

### 2.2. Linear B Cell Epitope Prediction

When exposed to B cell epitopes, B cell lymphocytes differentiate into memory and plasma cells, making them important from the standpoint of vaccine development. The ABCpred server was used to predict linear B cell epitopes (10 mers). The ABCpred server (https://webs.iiitd.edu.in/raghava/abcpred/ABC_submission.html, accessed on 10 February 2023) has an accuracy of 75% (0.75 specificity and 0.49 sensitivity) [27]. For subsequent analysis, only epitopes having a threshold of 0.51 or above were considered.

### 2.3. T Cell Epitope Prediction

#### 2.3.1. CTL Epitope Prediction

Cytotoxic T lymphocyte (CTL) epitopes are key components in the development of subunit vaccines. With a strong binder threshold of 0.5 percent and a weak binder threshold of 2 percent, the NetMHCpan 4.1 (https://services.healthtech.dtu.dk/service.php?NetMHCpan-4.1, accessed on 10 February 2023) tool was used to predict 9-mer CTL epitopes targeting the 12 HLA class I supertypes [28]. 

The IEDB class I immunogenicity server (http://tools.iedb.org/immunogenicity/, accessed on 10 February 2023) was utilized to further examine the immunogenicity of the identified CTL epitopes [29]. For the construct of multi-epitope vaccines, only epitopes with positive immunogenicity scores were used.

#### 2.3.2. HTL Epitope Prediction

The direction of both humoral and cellular immune responses is determined by HTL epitope prediction, which is a critical step in the development of preventive and immunotherapeutic vaccines. The Immune Epitopes and Analysis Resource (IEDB) server (http://tools.immuneepitope.org/mhcii/, accessed on 10 February 2023) was used to predict HTL epitopes [30]. The IEDB server is a freely accessible online database that stores experimentally tested immunogenic epitope data, sorting promiscuous binders by percentile rank and IC50 value, as well as host-specific alleles. In the case of MHC II, epitopes with an IC50 value less than 50 and the lowest percentile rank (lowest percentile rank implies strong binding affinity) were chosen for vaccine development.

### 2.4. Screened Epitopes Characterization

The antigenicity of the epitope sequences was predicted using VaxiJen v2.0 (http://www.ddg-pharmfac.net/vaxijen/VaxiJen/VaxiJen.html, accessed on 10 February 2023), with the accuracy of the prediction parameter set to 0.4 [31]. Furthermore, predicted epitopes were run on the AllerTop V 2.0 (https://www.ddg-pharmfac.net/AllerTOP/, accessed on 10 February 2023) server to remove allergic proteins [32]. AllerTop uses an alignment-free technique based on the key physicochemical features of proteins to predict allergens. The server sensitivity is approximately 94%. ToxinPred (http://crdd.osdd.net/raghava/toxinpred/, accessed on 10 February 2023) was also used to predict the toxicity of the selected epitopes. To evaluate toxicity, the support vector machine (SVM) approach was employed on the server, with all parameters set to their default settings. The SVM is a widely used machine-learning technique for toxicity prediction, because it can effectively distinguish between toxic and non-toxic epitopes [33].

### 2.5. Assembly of the Multi-Epitope Vaccine

Because of their smaller size, peptides are naturally non-immunogenic when used alone as a vaccine. They require a carriage containing a potent immune-stimulatory adjuvant in order to activate both the innate and adaptive immune systems. Linkers are also important for replicating the vaccine construct’s capacity to serve as an independent immunogen and to produce antibody concentrations larger than a single immunogen. The multi-epitope vaccine was developed by combining screened B cell and CTL and HTL epitopes with the help of KK, AAY, and GPGPG linkers, respectively. An adjuvant phenol-soluble modulin α4 was added to the N-terminus of the final vaccine using an EAAAK linker. The constructed vaccine was further checked for antigenic and non-allergenic properties.

### 2.6. Population Coverage Analysis of Vaccine Construct

A key prerequisite for producing a successful multi-epitope vaccine is the distribution of certain HLA alleles among diverse ethnicities and populations around the world. The expression of various HLA alleles varies across ethnicities worldwide. The population coverage of the best-selected epitopes across multiple HLA alleles was determined using the IEDB population coverage tool (http://tools.iedb.org/population/, accessed on 10 February 2023) [34]. These estimates are based on HLA genotypic frequencies, with non-linkage disequilibrium between HLA loci being assumed.

### 2.7. Physicochemical Features and Secondary Structure and Solubility Prediction of the Vaccine Construct

The ProtParam tool (https://web.expasy.org/protparam/, accessed on 10 February 2023) was used to analyze the physical and chemical properties of multi-epitope vaccines, including the amino acid composition, estimated half-life, instability index, extinction coefficient, theoretical pl, atomic composition, molecular weight, and grand average of hydropathicity (GRAVY) to help experimental studies [35]. The stability index is one of the most important parameters to consider, because it assists in the elimination of unstable protein candidates (protein instability index > 40).

PSIPRED (http://bioinf.cs.ucl.ac.uk/psipred/, accessed on 10 February 2023) was used to predict the secondary structure of the vaccine’s primary amino acid sequence [36]. It is based on two feed-forward neural networks that analyze the output of the Position-Specific Iterated–BLAST algorithm (PSI-BLAST).

To predict the propensity of protein solubility, the SOLpro (https://scratch.proteomics.ics.uci.edu/, accessed on 10 February 2023) server was used. This server predicts whether a protein will be soluble when overexpressed in E. coli using a two-stage SVM architecture based on several representations of the basic sequence. The first layer’s classifiers each accept a separate set of attributes as input to characterize the sequence. The data are summarized by a final SVM classifier, which predicts whether or not the protein is soluble, as well as the probability associated with its solubility [37].

### 2.8. Tertiary Structure Prediction and Validation of the Multi-Epitope Vaccine

The putative 3D structure of the fused protein vaccine was modelled using the Robetta server (https://robetta.bakerlab.org/, accessed on 10 February 2023) and AlphaFold server [38]. The Robetta server looks for templates that are suitable for comparative modeling and uses them. If a template isn’t available, however, the De-novo rosetta fragment insertion technique is used by the server. Meanwhile, the AlphaFold server is an AI system that predicts a protein’s 3D structure from its amino acid sequence. After that, the protein’s structure was validated using the online servers PROCHECK [39], ERRAT [40], and Verify 3D [41] (https://saves.mbi.ucla.edu/, accessed on 10 February 2023). The Ramachandran plot is used to forecast whether a certain amino acid would form a secondary structure based on dihedral angles ɸ and Ψ (allowed and disallowed) of amino acids determined using the Van der Waals radius of side chain atoms. The percentage of residues in the favored region, allowed region, and outlier region were used to assess the quality of the modeled tertiary structure. The ERRAT server was used for statistical analysis of nonbonded interaction. PyMOL (https://pymol.org/, accessed on 10 February 2023) was used to visualize the vaccine construct 3D structure.

### 2.9. Disulfide Engineering of the Vaccine Protein

The geometric conformation of proteins is enhanced by disulfide bonds, which provide significant stability. Disulphide by Design 2.13 (http://cptweb.cpt.wayne.edu/DbD2/index.php, accessed on 10 February 2023), an online server, was used to design such bonds for the constructed vaccine protein [42]. The server recognizes and delivers a list of residue pairs with proper geometry that can form disulfide bonds when individual amino acids are changed to cysteine.

### 2.10. Discontinuous B Cell Epitope Prediction

The ElliPro (http://tools.iedb.org/ellipro/, accessed on 10 February 2023) server was used to predict the final vaccine structure’s discontinuous B cell epitopes [43]. It assigns an ellipsoid score to each residue, which is defined as a PI (Protrusion Index) value based on the 3D structure of the protein antigen. With a PI of 0.9, the ellipsoid would include 90% of the protein residues, and the remaining 10% would be outside the ellipsoid. Greater-scoring residues are more solvent accessible and are grouped based on the distance R (between the residues’ centers of mass), with a higher R value suggesting more discontinuous epitopes.

### 2.11. Prediction of IFN-γ Inducing Epitopes

The IFNepitope (https://webs.iiitd.edu.in/raghava/ifnepitope/design.php/, accessed on 10 February 2023) server prediction module was used to anticipate the interferon gamma-triggering HTL epitopes [44]. This server was used to analyze the existence of the IFN epitope using all HTL peptides as input. Both motif and SVM hybrid algorithms were utilized as prediction algorithms. With its unique support vector machine (SVM) score, each input epitope output result was obtained.

### 2.12. Studies on Molecular Docking of Vaccine Constructs with TLR-2

To perform receptor-vaccine docking, RCSB (www.rcsb.org, accessed on 10 February 2023) was used to retrieve the 3D structure of the Toll-like receptor2 (TLR2) (PDB id: 3A7C). The ClusPro 2.0 (https://cluspro.bu.edu/login.php, accessed on 10 February 2023) server and HADDOCK (https://wenmr.science.uu.nl/, accessed on 10 February 2023) were used for protein receptor docking [45,46]. ClusPro is a docking approach that uses the pairwise RMSD histogram of all docked conformations to quickly classify docked conformations. Cluspro supports rigid body docking techniques such as DOT and ZDOCK, which are both based on the FFT correlation approach. HADDOCK is a popular docking program that takes a data-driven approach to dock, with support for a wide range of experimental data.

### 2.13. Molecular Dynamics Simulation

The purpose of using a molecular dynamics simulation is to examine the structural characteristics and microscopic connections between the receptor and the ligand in a docked complex [47]. The GROMACS-2019.4 software suite was used to perform molecular dynamics simulations in order to confirm the stability of the docked complex. In this case, sodium ions were solvated and added to the TIP3P water system for system neutralization. Prior to simulation, the steepest descent approach was employed to check that the system had no steric conflicts or unsuitable geometry, and the temperature was then raised to 300K. Following the equilibration process, the receptor–ligand complex was simulated on a 200 ns time scale. After the MD simulations for the bounded form of the target protein were completed, several Gromacs built-in functions were utilized to analyze various structural parameters such as the RMSD, RMSF, radius of gyration, and hydrogen bond.

### 2.14. MMPBSA Binding Free Energy Analysis

The binding free energies of the protein–protein complexes were calculated by the molecular mechanics-Poisson–Boltzmann solvent-accessible surface area, or the MMPBSA method, using the g_mmpbsa package. In this method, the binding free energy is the result of the free energy of the complex minus the sum of the free energies of the ligand and the protein. The MMPBSA calculation was performed for every ns of the simulation trajectory.

ΔGbind = Δgcomplex − (Δgligand + ΔGReceptor)(1)

### 2.15. Codon Adaptation and In Silico Cloning

The multi-epitope vaccine sequence was reverse-translated and then optimized for Escherichia coli codon use, resulting in higher expression of the multi-epitope vaccine sequence cloned in the mentioned expression system. The Java Codon Adaptation Tool (Jcat) server (http://www.jcat.de/, accessed on 10 February 2023) was used for the entire activity. The GC content and codon adaptation index (CAI) of cloned sequences were measured to assess their expression. A ratio of 0.8 to 1 CAI is deemed optimum due to favorable transcriptional and translational efficiency, whereas the proper GC content should range between 30–70 percent. SnapGene was used to clone the engineered construct into the pET-28a (+) expression vector.

### 2.16. Immune Simulation of Vaccine Constructs

To estimate vaccine immunogenicity and immune response profile, an immune simulation study was performed. The vaccine sequence was tested for its ability to mimic different immune cell types, such as T helper and T cytotoxic cells, B cells, NK cells, macrophages, dendritic cells, immunoglobulins, and cytokines. With the exception of the time steps, which were set to 1, 84, and 168, and the number of simulation steps, which were set to 1050, the remainder of the parameters were left at their default levels for the experiment.

## 3. Results

### 3.1. Epitope Prediction for B Cell Lymphocytes

For the identification of linear B cell epitopes, the ABCPred server was employed. The amino acid length was set at 10, and the score threshold was set at 0.51 for this prediction. Among all the 31 predicted epitopes from the CnBP protein, the two peptides (LKFMVFIMLL and KDNQDGKRPE) of 10mer that had the highest antigenicity score were selected after the epitopes were characterized based on antigenicity, allergenicity, and toxicity. (Table 1). With a prediction accuracy of 65.9%, the ABCPred server comprises epitopes from various species, such as viruses, bacteria, and parasites (Supplementary Table S1). 

### 3.2. Epitope Prediction for T Cells

#### 3.2.1. Epitope Prediction for Cytotoxic T-Lymphocyte (CTL)

The NetMHCpan 4.1 server was used to identify the CTL epitopes in the protein. From all 12 MHC-I supertypes, a total of 120 antigenic, non-allergenic, and non-toxic CTL epitopes (9-mer) were predicted (Supplementary Table S2). The class I immunogenicity tool on the IEDB server was also used to examine the immunogenicity and tendency of activating cytotoxic T cells for the identified MHC class I binding epitopes. Among all the predicted epitopes, only 10 CTL epitopes with the highest antigenicity score and a positive immunogenicity score (TPDGATITF, KVNGYTTHV, TTPEGYTKK, KFMVFIMLL, KPTIYFKLY, EFEVQGRNL, VKPTIYFKL, TKNTIDVTI, IEYTVTEDH, and LLNIITPLF) were considered for the vaccine construct (Table 2). 

#### 3.2.2. Epitope Prediction for Helper T-Lymphocyte (HTL)

The development of preventive and immunotherapeutic vaccines requires the prediction of HTL epitopes. They are important members of the adaptive immune system, as T cells release cytokines that govern nearly all adaptive immunological responses. The HTL epitopes were predicted for a reference panel of 27 alleles using the IEDB server. Out of many epitopes predicted (Supplementary Table S3), the two best 15mer epitopes were selected that exhibited the highest antigenic score and at the same time had an IC50 value of less than 50 (peptides with a binding affinity of less than 50 nM are regarded as having the maximum binding affinity), which were also non-allergenic and non-toxic. Based on the aforementioned criteria, the final two HTL epitopes (NVLKFMVFIMLLNII and VLKFMVFIMLLNIIT) were selected from 10 predicted epitopes (Table 3).

### 3.3. Designing Multi-Epitope Subunit Vaccine Construct

**Figure 2:**
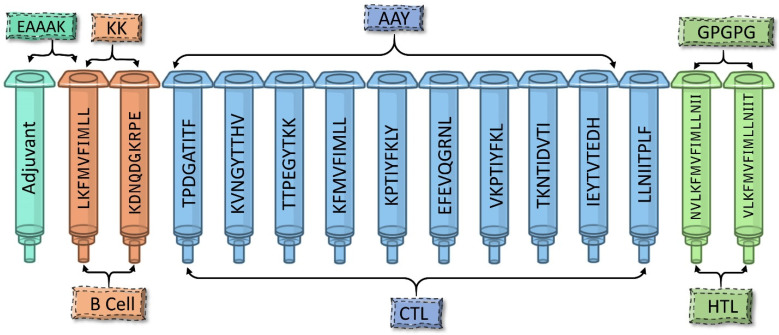
The structural arrangement of B and T cell epitopes along with linkers and adjuvant for the final multi-epitope vaccine construct.

The multi-epitope vaccine was created by combining epitopes from B cells, CTLs, and HTLs. With the help of various linkers, antigenic epitopes from B cells, CTLs, and HTLs were all connected (Figure 2). In order to provide amino acid residues with the most flexibility, linkers are essential for prolonged conformation or protein folding. B cell epitopes use the KK linker, CTL epitopes use the AAY linker, and HTL epitopes use the GPGPG linker. An effective, immunogenic, and well-tolerated adjuvant is required to improve the immunogenicity of the prepared vaccine. It is reported that phenol-soluble modulin (PSM) α4 mediates HBP (heparin-binding protein) from the release of polymorphonuclear neutrophils (PMNs) during *S. aureus* infection-induced vascular leakage [48]. The strong impact that PSMα peptides have on the progress of acute forms of various *S. aureus* disease identifies them as promising targets for drug development [49]. Henceforth, phenol-soluble modulin α4 (20 amino acids’ length) was added as an adjuvant to the construct’s N-terminus, along with an EAAAK helix-forming linker. The vaccine’s final construct consisted of 207 amino acid residues.

### 3.4. Allergenicity and Antigenicity Prediction of the Vaccine Construct

The AllerTOP v.2.0 server was used to determine the allergenicity of the vaccine construct. According to the results, the vaccine was non-allergenic in nature. The antigenicity of the construct was also predicted using VaxiJen v2.0. The predicted antigenicity was 0.8725, indicating that the vaccine’s antigenicity was significant. As a result, we came to the conclusion that the vaccine construct was an effective antigen.

### 3.5. Prediction of Population Coverage

MHC molecules’ peptide-binding regions are extremely polymorphic; they have a wide range of binding specificity. Furthermore, the distribution of HLA alleles varies across different geographic regions and ethnic groups around the world [50]. As a result, the population coverage was estimated using 10 CTL epitopes and two HTL epitopes and their corresponding HLA alleles (which encompasses 109 countries covering 16 different geographical regions). As the vaccine protein is comprised of both CTL and HTL epitopes, we focused on the combined T cell coverage, which covers 99.51% of the global population (Supplementary Table S4).

### 3.6. Physicochemical Characterization, Secondary Structure, and Solubility of Designed Vaccine Construct

The final vaccine molecular weight was calculated to be 23.68 kDa. The vaccine was demonstrated as a small-scale construct that is simple to handle and purify during testing. Due to its 9.46 theoretical pI value, the vaccine candidate was discovered to be slightly basic in nature. There are 13 negatively charged amino acid residues and 22 positively charged amino acid residues in this protein. In mammalian cells, the vaccine had a half-life of 30 hours; it was more than 20 hours in yeast cells and more than 10 hours in *E. coli* cells. The constructed vaccine’s instability index (II) is 72.73, denoting that it is extremely stable. The protein aliphatic index (AI) determines the vaccine’s thermal stability; a score of 108.50 indicates that it is thermally stable, and the grand average of hydropathicity (GRAVY) was 0.502 (Supplementary Table S5).

PSIPRED was used to predict the final subunit vaccine construct’s secondary structure (Supplementary Figure S1). Alpha helices contain 53.14 percent of the sequence, beta strands contain 25.12 percent, and random coils contain the remaining 21.73 percent. These findings indicate the possibility of inter-chain and intra-chain interactions in the creation of the protein’s secondary structure. Solubility is a crucial component in post-production vaccine research, since more solubility indicates better purification during downstream processing [37]. When the vaccine protein was over-expressed in *E. coli*, the SolPro servers discovered that it was soluble (Supplementary Table S5).

### 3.7. Prediction and Validation of Tertiary Structure

**Figure 3:**
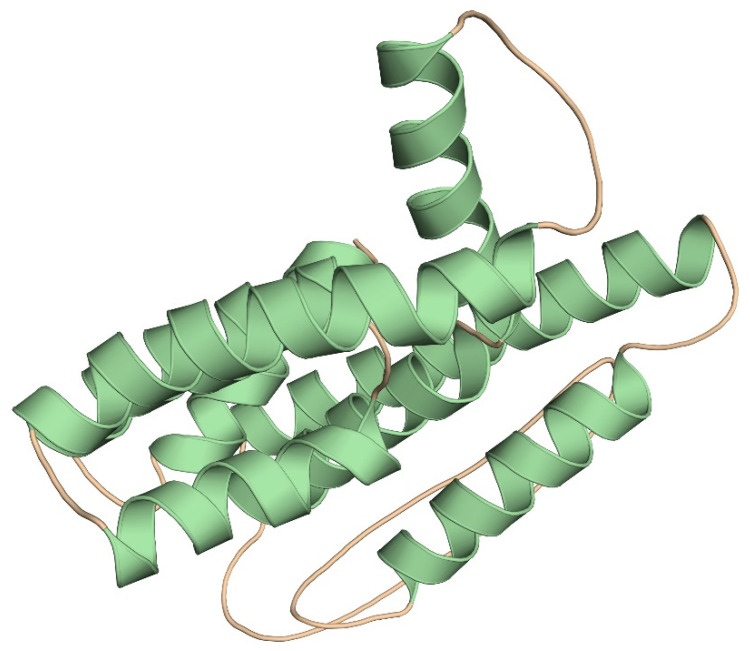
Homology modeling of the three-dimensional structure of the final multi-epitope vaccine construct.

The Robetta server and Alphafold were used to develop the tertiary structure of the subunit vaccine protein for its functional characterization. Models from both the servers were chosen for further investigation after careful consideration (Figure 3). We used the PROCHECK, ERRAT, and Verify 3D servers to identify and correct potential errors in the predicted 3D structure. In the predicted 3D model, the Ramachandran plot analysis showed 92.3 percent, 6.0 percent, and 0.5 percent of residues were found in the favored, additionally allowed, and generously allowed regions, respectively, and only two residues were found in the disallowed regions (Supplementary Figure S2). The ERRAT was also used to verify the quality of the modeled structure. According to the results, the overall quality factor of the three-dimensional model was 97.487. Furthermore, the Verify 3D score was 83.09, suggesting that more residues were located in acceptable side chain regions (Supplementary Figure S2). Further validation of the modeled structure revealed that it was of decent quality and had a high level of stability, and it was then subjected to docking analysis.

### 3.8. Vaccine Protein Disulfide Engineering

**Figure 4:**
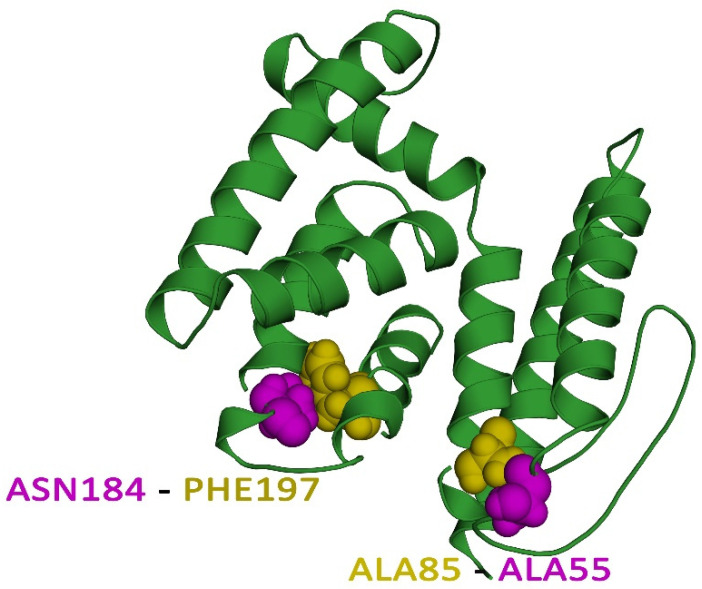
Disulphide engineering of the vaccine protein. Residue pairs shown in purple (ALA55, ASN184), and olive (ALA85, PHE197) spheres were mutated to cysteine residues to form a disulphide bridge between them.

Protein stability is vital in a wide range of biological applications, and it is an enticing method to replicate nature’s molecular interaction stabilization [51]. The covalent disulfide bonds give target proteins much stability, and disulfide engineering has had a lot of success in many different applications [52]. For the purpose of disulfide engineering, two pairs of residues were chosen, ALA55-ALA85 and ASN184-PHE197, based on the criteria that the bond energy score should be fewer than 2.2 kcal/mol, and the χ3 value should be between from −87 to +97°. Figure 4 shows the disulfide engineering of the multi-epitope vaccine structure (Supplementary Table S6).

### 3.9. Prediction of Discontinuous B Cell Epitopes

**Figure 5:**
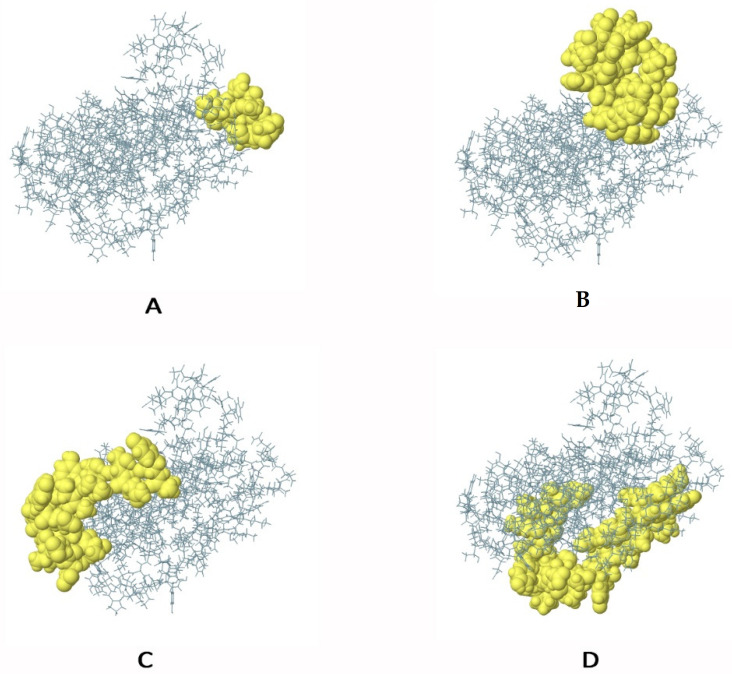
The conformational B-lymphocyte epitopes present in the vaccine. The yellow spheres showing epitopes containing (**A**) 10 residues (AA 23-32) with 0.836; (**B**) 24 residues (AA 108-131) with 0.806; (**C**) 28 residues (AA 133-134, AA 137-138, AA 141-142, AA 144-157, AA 182-183, and AA 185-190) with 0.718; (**D**) 40 residues (AA 1-6, AA 9, AA 50-56, and AA 58-83) with 0.655.

The ElliPro software revealed four conformational B cell epitopes in the constructed vaccine protein, as shown in Figure 5. As shown in Supplementary Table S7, the epitopes ranged in size from 10 to 40 residues, with a total of 102 residues. Furthermore, the conformational B cell epitopes had scores ranging from 0.655 to 0.836.

### 3.10. IFN-γ Inducing Epitope Analysis

When naive HTLs are activated by APCs, T cells are differentiated into Th1 or Th2 helper cells, which secrete cytokines. When Th1 cells are differentiated, IFN-γ is released, which is a type II interferon. In both the innate and adaptive immune responses, Th1 cells release IFN-γ. This causes macrophages to become activated, which are important for controlling intracellular pathogens. All the selected HTL epitopes (NVLKFMVFIMLLNII and VLKFMVFIMLLNIIT) were shown to be effective at inducing IFN-γ production.

### 3.11. Molecular Docking of the Designed Vaccine with Immunological Receptor

**Figure 6:**
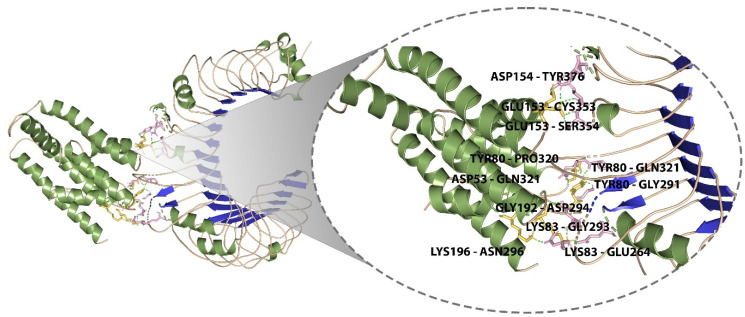
Molecular interaction of multi-epitope vaccine construct docked with TLR2.

TLR2 plays a key role in staphylococcal illness by regulating the stimulation of polymorphonuclear neutrophils, the expression of adhesion molecules, chemotaxis, and the expression of chemoattractant receptors, as well as the detection of ligands and phagocytosis, among other professional phagocyte functions. Lipoproteins appear to play a key role in TLR2 activation by staphylococci, according to growing evidence. Therefore, TLR2 was chosen as the immune receptor [53,54,55,56]. Molecular docking was done between the final vaccine design and the TLR2 immune receptor (PDB ID:3A7C) using the freely accessible website ClusPro and HADDOCK. The best docked complex was chosen from both the servers based on the consensus approach. The model with the lowest binding energy was −1055, and the model with the best center energy between the ligand and the receptor was −969. Pymol was used to visualize the interactions between the TLR2 receptor and the vaccine candidate, and the docked complex is shown in Figure 6. Residues of the vaccine construct and the TLR2 receptor that were found to make polar contacts included ASP154-TYR376, GLU153-SER354, GLU153-CYS353, LYS196-ASN296, GLY192-ASP294, LYS83-GLU264, LYS83-GLY293, TYR80-GLY291, TYR80-GLN321, TYR80-PRO320, and ASP53-GLN321 with a distance of 1.9 Å, 1.8 Å, 2.3 Å, 1.7 Å, 1.9 Å, 2.2 Å, 1.7 Å, 2.0 Å, 1.9 Å, 1.9 Å, and 2.0 Å, respectively.

### 3.12. Molecular Dynamics Simulation

**Figure 7:**
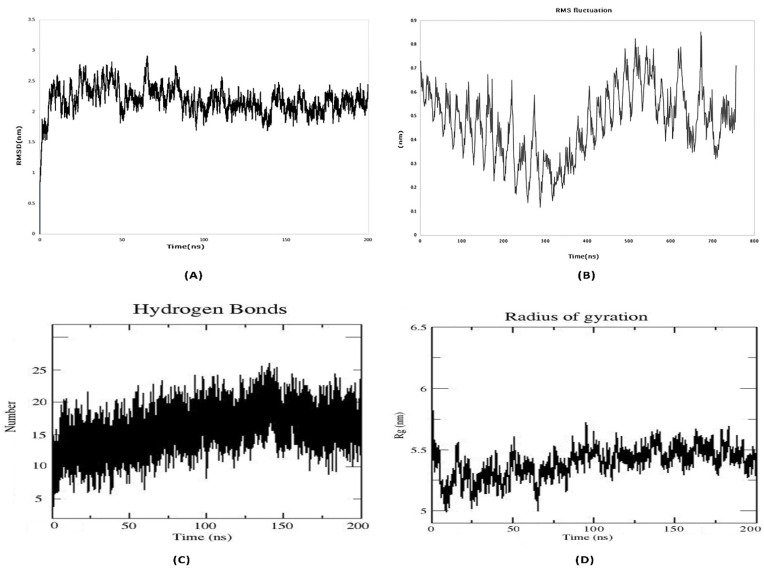
Molecular dynamics simulation study of TLR2-vaccine complex representing. (**A**) Root Mean Square Deviation, (**B**) Root Mean Square Fluctuation (**C**) Radius of gyration, (**D**) Number of hydrogen bonds formed during MD simulation.

For the MD simulation, the best molecular docking complex was employed as an input. At this point, an MD simulation of the complex was run for 200 ns, and the results were evaluated in terms of the root mean square deviation (RMSD), the root mean square fluctuation (RMSF), the number of hydrogen bonds (H-bonds) and the radius of gyration (Rg). The alterations in a protein’s backbone, from its initial structural conformation to its end point, are displayed in the RMSD profile. The average RMSD value of the receptor–vaccine combination, which was computed for all of the backbone C atoms, was 2.5 nm (Figure 7A). Consequently, a stable vaccine–receptor complex may emerge as a result of favorable interactions. The RMSF provides details regarding a protein’s residue-by-residue dynamics in relation to its beginning position. To ascertain the conformational behavior of the ligand receptor complex at the residue level, the RMSF of the Cα atoms was examined. The average RMSF reading was 0.5 nm (Figure 7B). In both the stabilization of the protein structure and the identification of ligands by proteins, hydrogen bond interactions are crucial. In order to better understand the specificity of interactions and the potential for selective intermolecular interactions, the number of hydrogen bonds that were created in the vaccine receptor complex throughout the MD simulations were also examined. The vaccine–receptor complex generated an average of 15-20 hydrogen bonds (Figure 7C). The average radius of gyration (Rg) for the vaccine–TLR-2 complex was found to be 5.25 nm during the simulation (Figure 7D). These findings showed that the compactness of the complex increased following a favorable interaction between the vaccine protein and the TLR-2 receptor.

### 3.13. MMPBSA Binding Free Energy Analysis

The free energy of binding (ΔG_bind_) is believed to be an essential thermodynamic quantity to assess the favorable protein–protein interaction, as well as the affinity for accurate modeling of biological systems. In this regard, the g_mmpbsa tool was used to calculate the free binding energy of the MD simulations. MM/PBSA calculates the aforementioned favorable forces, including the solvent-accessible surface area (SASA) and unfavorable polar solvation energy (PSE). The MM/PBSA computed free energy of binding for the system was estimated as -109.941 ± 15.971 kJ/mol (Table 4). The results revealed that the docking was energetically feasible, as indicated by the negative values of Gibbs free energy (ΔG). The negative free binding energy value observed indicates that the vaccine complex strongly binds to the receptor, making it a promising candidate as a vaccine against *S. aureus*.

### 3.14. Codon Optimization and In Silico Cloning

**Figure 8:**
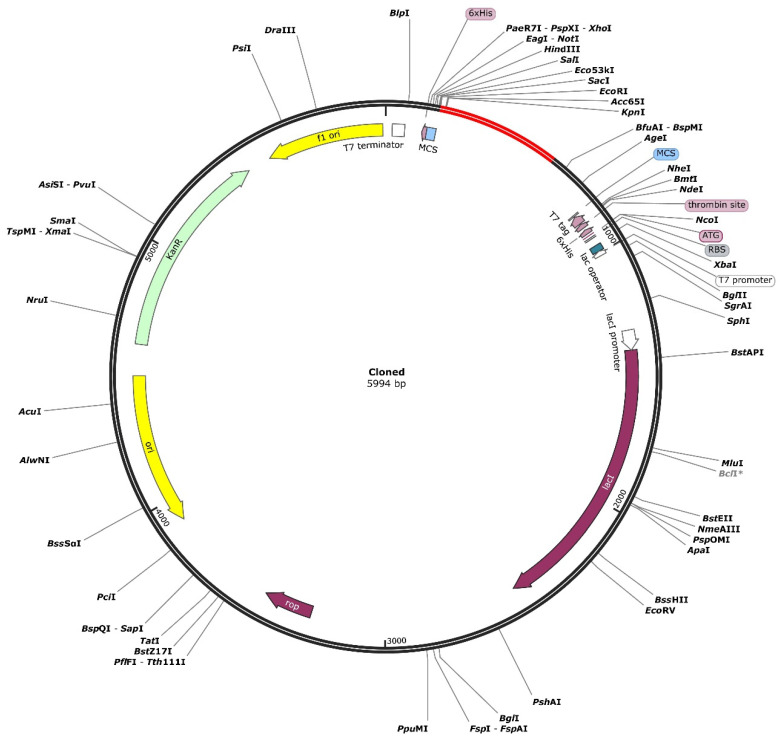
Restriction cloning of final multi-epitope vaccine using pET28a (+) expression vector in the in silico space. Black circle indicates the vector, and the red part is the place in which the vaccine is inserted.

It is crucial to know whether such multi-epitopic vaccines can be cloned and expressed in an appropriate expression vector. Thus, in silico cloning was utilized to examine the cloning and expression efficiency of the vaccine construct in an expression vector. In the vector *E. coli* (strain K12), the codon sequence was improved utilizing the Java Codon Adaptation Tool (JCat). It was 621 nucleotides long, with a GC content of 47.02 percent and a Codon Adaptation Index (CAI) of 0.99. Further, 6x histidine was tagged to facilitate purification and solubilization of the vaccine protein after expression. The results show that the suggested vaccine design may be expressed efficiently in the *E. coli* K12 (host) expression vector. The multi-epitope vaccine-adapted codon sequence was also inserted into the pET28a (+) vector using the SnapGene tool for restriction cloning (Figure 8).

### 3.15. Immune Simulation

**Figure 9:**
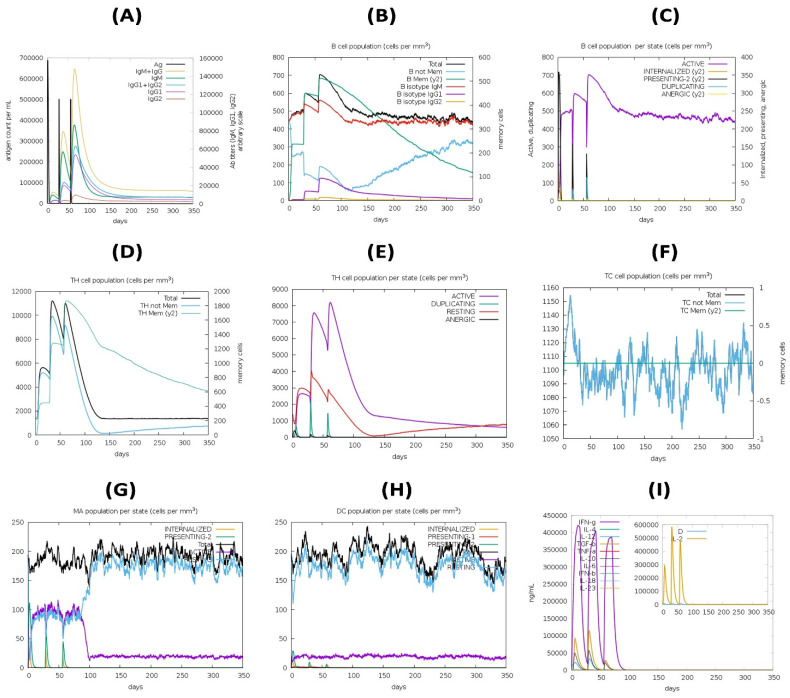
In silico simulation of immune response using vaccine as antigen: (**A**) antigen and immunoglobulins, (**B**) B cell population, (**C**) B cell population per state, (**D**) helper T cell population, (**E**) helper T cell population per state, (**F**) cytotoxic T cell population per state, (**G**) macrophage population per state, (**H**) dendritic cell population per state, and (**I**) production of cytokine and interleukins with Simpson index (D) of immune response.

The secondary and tertiary responses were higher than the primary response (Figure 9). Immunoglobulin (Ig) M concentrations were found to be significantly higher than IgG concentrations. Both secondary and tertiary responses displayed typical high levels of immunoglobulin activity (i.e., IgG1 + IgG2, IgM, and IgG + IgM antibodies) with the antigen reduction. This shows the development of immunological memory and, as a result, efficient antigen immunity in repeated antigen exposures. Furthermore, several B cell isotypes were found to exist for a long time, implying the possibility of isotype switching and memory formation. With their respective memory development, the CTL and HTL populations showed a similar elevated response. Additionally, macrophage activity increased, although dendritic cell activity remained constant. IFN-γ and IL-2 levels were also elevated. In addition, components of the innate immune system (such as epithelial cells) were active. The probability of a wide variety of immunological responses was also suggested by a lower Simpson index (D) (Figure 9).

## 4. Discussion

*Staphylococcus aureus* is a major cause of morbidity and mortality around the world [57,58]. It is especially well known as a dangerous hospital-acquired pathogen. Furthermore, many *S. aureus* strains are resistant to a wide range of other antibiotics, leaving only a few treatment options [59,60]. In an era in which pharmaceutical companies have largely abandoned the development of much-needed new antibiotics, researchers in the industry and academia are once again attempting to develop vaccines against *S. aureus* [61]. There is no effective vaccine against *S. aureus* infections. CnBP, a collagen (Cn)-binding protein, is involved in bacterium–host adherence as well as immune evasion [5]. The CnBP protein was used to develop a vaccine that elicited both protective humoral and cellular responses, resulting in protection against various *S. aureus* infections. Traditional vaccine development techniques introduced by Louis Pasteur are widely used [62], but they have a number of drawbacks, including side effects, difficulty in development, and cost [62,63,64,65,66]. Whole-cell vaccines are less immunogenic and might not be efficient at separating the particular antigen. Epitope-based vaccines are currently being developed with promising results to provide both preventive and therapeutic effects on pathogen-specific immunity. This strategy has various advantages, including the elimination of undesirable immune responses by designing specific constructs; (i) the generation of prolonged immunity with required responses; and (ii) cost and time effectiveness. The low immunogenicity of single-epitope peptides led to the idea of designing constructs with multiple epitopes. Computational vaccine development is a feasible alternative to traditional vaccine development because of its specificity, safety, stability, and cost effectiveness [67].

The current study uses immunoinformatics and structural investigations to identify potential T cell and B cell epitopes in order to design a novel vaccine with a rational approach. To design a peptide vaccine against the CnBP protein, T cell and B cell epitopes were identified. The existence of IFN-gamma-inducing epitopes was checked in the final vaccine design, in addition to the prediction of HTL epitopes; the chosen epitopes were found to be IFN-gamma-inducing epitopes. To develop the vaccine construct, the targeted T and B cell epitopes were fused utilizing the KK, AAY, and GPGPG linkers, respectively. Due to the limited number of epitopes involved, subunit vaccines have a poor immunogenicity; however, this can be enhanced with the addition of adjuvant. To create a vaccine, phenol-soluble modulin α4 was added at the N-terminus using the EAAAK linker.

Antigenic and non-allergenic property predictions indicated the vaccine’s effectiveness and safety [64,68,69]. The vaccine protein was discovered to be antigenic with a score of 0.8725. The final vaccine was discovered to be 207 amino acids long, with a non-allergenic construct that has the ability to trigger an antigenic response. Chimeric protein physicochemical parameters were investigated. Importantly, the designed vaccine was discovered to be highly soluble, making purification a breeze. The vaccine’s secondary and tertiary structure was generated by PSIPREDV3.3 and the Robetta server. Alpha helices were found in a higher percentage of secondarily designed vaccine constructs (53.14%), with 25.12% and 21.73% of extended strand and random coils, respectively. In order to detect errors in the 3D model, validation servers were used. The Ramachandran plot was evaluated using the Procheck server. The majority of vaccine residues, according to Procheck, fell in the most favorable region.

TLR agonists have proven to be effective adjuvants in recent years, with several of them already being used in approved vaccines [70,71]. ClusPro 2.0 was used to conduct molecular docking studies to investigate the affinity of binding interactions between the chimeric vaccine and TLR2. There were, on average, 11 H-bonds between the receptor and the vaccine complex. The vaccine and the receptor protein (TLR2) had a chance to attain their most stable conformations with respect to each other throughout the dynamic MD simulation evaluation. The vaccine has been cloned into the pET28a (+) vector for expression in a bacterial medium for ease of production and purification. However, before the vaccine can be used in the general public, it must be tested in real-time in vitro experiments. In essence, the multi-peptide sub-unit vaccine has enormous potential to serve as an alternative vaccine and to alleviate the *S. aureus* infection crisis.

## 5. Conclusions

The current study presents a new multi-epitope vaccine against *Staphylococcus aureus*. A combination of immunoinformatics analysis, protein structure evaluation, and physicochemical and molecular dynamics analyses all validated the suggested vaccine’s ability to elicit a significant humoral and cellular immunological response, providing compelling evidence for future experimental validation.

## Figures and Tables

**Table 1:** Final selected B cell epitopes from *S. aureus* collagen-binding protein and their corresponding immunogenic properties.

Uniprot_ID	B Cell Epitope	Position	Score	Antigencity Score	Toxicity	Hydrophobicity	Hydropathicity	Hydrophilicity	Charge	Mol wt.
**Q53654**	LKFMVFIMLL	6	0.69	2.1966	NON-TOXIN	0.35	2.56	−1.33	1	1254.86
	KDNQDGKRPE	546	0.67	2.3986	NON-TOXIN	−0.73	−3.18	1.84	0	1186.38

**Table 2:** Predicted CTL epitopes from *S. aureus* collagen-binding protein to design multi-epitope vaccine construct with their corresponding MHC class I alleles and their immunogenic properties.

Uniprot_ID	CTL Epitope	Alleles	Position	Score	Antigencity Score	Immunogenicity	Toxicity	Hydrophobicity	Hydropathicity	Hydrophilicity	Charge	Mol wt.
**Q53654**	TPDGATITF	HLA-A*01:01, HLA-A*02:01, HLA-A*03:01, HLA-A*24:02, HLA-A*26:01, HLA-B*07:02, HLA-B*08:01, HLA-B*27:05, HLA-B*39:01, HLA-B*40:01, HLA-B*58:01, HLA-B*15:01	107	1.279	0.9339	0.25094	NON-TOXIN	0.05	0.17	−0.33	−1	922.12
	KVNGYTTHV	HLA-A*02:01, HLA-A*01:01, HLA-A*03:01, HLA-A*24:02, HLA-A*26:01, HLA-B*07:02, HLA-B*08:01, HLA-B*27:05, HLA-B*39:01, HLA-B*40:01, HLA-B*58:01, HLA-B*15:01	1069	0.226	1.5081	0.1166	NON-TOXIN	−0.14	−0.59	−0.38	1.5	1018.27
	TTPEGYTKK	HLA-A*03:01, HLA-A*01:01, HLA-A*02:01, HLA-A*24:02, HLA-A*26:01, HLA-B*07:02, HLA-B*08:01, HLA-B*27:05, HLA-B*39:01, HLA-B*40:01, HLA-B*58:01, HLA-B*15:01	511	0.996	0.8267	0.03343	NON-TOXIN	−0.36	−1.86	0.61	1	1024.26
	KFMVFIMLL	HLA-A*24:02, HLA-A*01:01, HLA-A*02:01, HLA-A*03:01, HLA-A*26:01, HLA-B*07:02, HLA-B*08:01, HLA-B*27:05, HLA-B*39:01, HLA-B*40:01, HLA-B*58:01, HLA-B*15:01	7	0.635	1.7858	0.06914	NON-TOXIN	0.33	2.42	−1.28	1	1141.68
	KPTIYFKLY	HLA-B*07:02, HLA-A*01:01, HLA-A*02:01, HLA-A*03:01, HLA-A*24:02, HLA-A*26:01, HLA-B*08:01, HLA-B*27:05, HLA-B*39:01, HLA-B*40:01, HLA-B*58:01, HLA-B*15:01	448	2.837	1.6477	0.06464	NON-TOXIN	−0.06	−0.18	−0.57	2	1172.56
	EFEVQGRNL	HLA-B*08:01, HLA-A*01:01, HLA-A*02:01, HLA-A*03:01, HLA-A*24:02, HLA-A*26:01, HLA-B*07:02, HLA-B*27:05, HLA-B*39:01, HLA-B*40:01, HLA-B*58:01, HLA-B*15:01	130	1.717	1.6709	0.03304	NON-TOXIN	−0.28	−0.9	0.4	−1	1091.32
	VKPTIYFKL	HLA-B*27:05, HLA-A*01:01, HLA-A*02:01, HLA-A*03:01, HLA-A*24:02, HLA-A*26:01, HLA-B*07:02, HLA-B*08:01, HLA-B*39:01, HLA-B*40:01, HLA-B*58:01, HLA-B*15:01	447	3.894	1.6698	0.13438	NON-TOXIN	0	0.43	−0.48	2	1108.52
	TKNTIDVTI	HLA-B*39:01, HLA-A*01:01, HLA-A*02:01, HLA-A*03:01, HLA-A*24:02, HLA-A*26:01, HLA-B*07:02, HLA-B*08:01, HLA-B*27:05, HLA-B*40:01, HLA-B*58:01, HLA-B*15:01	257	0.65	0.9440	0.24496	NON-TOXIN	−0.11	0.02	−0.01	0	1004.28
	IEYTVTEDH	HLA-B*40:01, HLA-A*01:01, HLA-A*02:01, HLA-A*03:01, HLA-A*24:02, HLA-A*26:01, HLA-B*07:02, HLA-B*08:01, HLA-B*27:05, HLA-B*39:01, HLA-B*58:01, HLA-B*15:01	969	2.678	1.1265	0.21206	NON-TOXIN	−0.16	−0.86	0.23	−2.5	1106.28
	LLNIITPLF	HLA-B*15:01, HLA-A*01:01, HLA-A*02:01, HLA-A*03:01, HLA-A*24:02, HLA-A*26:01, HLA-B*07:02, HLA-B*08:01, HLA-B*27:05, HLA-B*39:01, HLA-B*40:01, HLA-B*58:01	14	0.567	0.6552	0.28212	NON-TOXIN	0.31	1.93	−1.3	0	1043.46

**Table 3:** Predicted HTL epitopes from *S. aureus* collagen-binding protein to design multi-epitope vaccine construct with their corresponding MHC class II alleles and their immunogenic properties.

Uniprot_ID	MHC II Epitope	Alleles	Pos	IC50 Value	Percentile_Rank	Antigencity Score	Toxicity	Hydrophobicity	Hydropathicity	Hydrophilicity	Charge	Mol wt.
**Q53654**	NVLKFMVFIMLLNII	HLA-DPA1*01:03, HLA-DPB1*02:01, HLA-DPB1*01:01, HLA-DRB1*01:01, HLA-DRB1*09:01,HLA-DRB3*02:02, HLA-DRB1*13:02, HLA-DRB1*11:01, HLA-DRB1*04:01, HLA-DRB1*12:01, HLA-DPA1*03:01, HLA-DPB1*04:02, HLA-DRB1*04:05, HLA-DRB1*15:01, HLA-DQA1*01:01, HLA-DQB1*05:01, HLA-DRB1*08:02, HLA-DPA1*02:01, HLA-DPB1*14:01, HLA-DPB1*04:01, HLA-DQA1*05:01, HLA-DQB1*03:01, HLA-DQA1*04:01, HLA-DQB1*04:02, HLA-DPA1*02:01, HLA-DPA1*02:01, HLA-DPB1*05:01, HLA-DPA1*01:03, HLA-DQA1*05:01, HLA-DQB1*02:01, HLA-DQA1*03:01, HLA-DQB1*03:02, HLA-DQA1*01:02, HLA-DQB1*06:02, HLA-DRB3*01:01, HLA-DRB5*01:01, HLA-DRB1*07:01, HLA-DRB4*01:01, HLA-DRB1*03:01	4–18	36	0.21	1.2706	NON-TOXIN	0.28	2.12	−1.2	1	1808.61
	VLKFMVFIMLLNIIT	HLA-DRB1*15:01, HLA-DRB3*01:01, HLA-DPA1*03:01, HLA-DPB1*04:02, HLA-DPA1*01:03, HLA-DPB1*02:01,HLA-DRB1*01:01, HLA-DRB1*09:01,HLA-DRB3*02:02, HLA-DRB1*13:02, HLA-DRB1*11:01, HLA-DRB1*04:01, HLA-DRB1*12:01,HLA-DRB1*04:05, HLA-DQA1*01:01, HLA-DQB1*05:01, HLA-DRB1*08:02, HLA-DPA1*02:01, HLA-DPB1*14:01, HLA-DPA1*01:03, HLA-DPB1*04:01, HLA-DQA1*05:01, HLA-DQB1*03:01, HLA-DQA1*04:01, HLA-DQB1*04:02, HLA-DPA1*02:01, HLA-DPB1*01:01, HLA-DPA1*02:01, HLA-DPB1*05:01,HLA-DQA1*05:01, HLA-DQB1*02:01, HLA-DQA1*03:01, HLA-DQB1*03:02, HLA-DQA1*01:02, HLA-DQB1*06:02, HLA-DRB5*01:01, HLA-DRB1*07:01, HLA-DRB4*01:01, HLA-DRB1*03:01	5–19	50	0.42	1.3372	Non toxin	0.31	2.31	−1.24	1	1795.61

**Table 4:** MM/PBSA free energy calculations of docked complex.

Energies	Energies (KJ/mol)	Std. Dev (KJ/mol)
van der Waal energy	−179.773	+/−56.735
Electrostatic energy	−26.076	+/−17.625
Polar solvation energy	116.241	+/−34.842
SASA energy	−20.333	+/−3.782
Total Binding energy	−109.941	+/−15.971

## Data Availability

The datasets generated during and/or analyzed during the current study are all present within the article and Supplementary Material.

## Supplementary Materials

The following supporting information can be downloaded at: https://www.mdpi.com/article/10.3390/pathogens12030376/s1, Figure S1: Secondary structure prediction of the final multi-epitope vaccine construct by using PSIPRED tool; Figure S2: Several structure validations tools results confirmed the modeled multi-epitope vaccine structure to be reliable and accurate; Table S1: Predicted B-cell epitopes epitopes from *S. aureus* Collagen binding protein and their corresponding immunogenic properties. The final selected epitopes to design multi-epitope vaccine construct from proteins has been highlighted in green colour; Table S2: Predicted CTL epitopes from *S. aureus* Collagen binding protein with their corresponding MHC Class I alleles and their immunogenic properties. The final selected epitopes to design multi-epitope vaccine construct from proteins has been highlighted in green colour; Table S3: Predicted HTL epitopes from *S. aureus* Collagen binding protein with their corresponding MHC Class II alleles and their immunogenic properties. The final selected epitopes to design multi-epitope vaccine construct from proteins has been highlighted in green colour; Table S4: Population Coverage analysis of the final vaccine construct using the population coverage analysis tool of the IEDB database by keeping the default parameters on (109 countries covering 16 different geographical regions); Table S5: Physicochemical Properties, secondary structure, and solubility analysis of final vaccine construct as predicted by ProtParam tool, PSIPRED, and SOLpro server; Table S6: Disulfide engineering of the final multi-epitope vaccine; Table S7: iscontinuous B-cell epitopes with their scores predicted by ElliPro.
