# Supplementary material for: Designing a Next-Generation Multiepitope-Based Vaccine against Staphylococcus aureus Using Reverse Vaccinology Approaches
Source: Pathogens. 2023 Feb 25;12(3):376. doi: 10.3390/pathogens12030376 (PMC10058999; doi:10.3390/pathogens12030376)
Supplement: Supplementary file 1 [file pathogens-12-00376-s001.zip › Supplementary Table S5.pdf]

Supplementary Table S5. Physicochemical Properties, secondary structure, and solubility analysis of final vaccine construct as predicted by ProtParam tool, PSIPRED, and SOLpro server.

<table>
<tr><th>Parameters</th><th>Vaccine Construction Sequence</th></tr>
<tr><td>Number of amino acids</td><td>207</td></tr>
<tr><td>Molecular weight</td><td>23.68 KDa</td></tr>
<tr><td>Theoretical pI</td><td>9.46</td></tr>
<tr><td>Total number of negatively charged residues (Asp + Glu)</td><td>13</td></tr>
<tr><td>Total number of positively charged residues (Arg + Lys)</td><td>22</td></tr>
<tr><td>Instability index</td><td>12.73 (Stable)</td></tr>
<tr><td>Aliphatic index</td><td>108.50</td></tr>
<tr><td>Grand average of hydropathicity (GRAVY)</td><td>0.502</td></tr>
<tr><td>The estimated half-life</td><td>30 hours (mammalian reticulocytes, in vitro).
>20 hours (yeast, in vivo)
>10 hours (Escherichia coli, in vivo).</td></tr>
<tr><td>Extinction coefficients</td><td>23840</td></tr>
<tr><td>Amino acid composition</td><td><table>
<tr><td>Ala (A)</td><td>27</td><td>13.0%</td></tr>
<tr><td>Arg (R)</td><td>2</td><td>1.0%</td></tr>
<tr><td>Asn (N)</td><td>8</td><td>3.9%</td></tr>
<tr><td>Asp (D)</td><td>6</td><td>2.9%</td></tr>
<tr><td>Cys (C)</td><td>0</td><td>0.0%</td></tr>
<tr><td>Gln (Q)</td><td>2</td><td>1.0%</td></tr>
<tr><td>Glu (E)</td><td>7</td><td>3.4%</td></tr>
<tr><td>Gly (G)</td><td>12</td><td>5.8%</td></tr>
</table></td></tr>
</table>

	His (H)	2	1.0%
	Ile (I)	24	11.6%
	Leu (L)	17	8.2%
	Lys (K)	20	9.7%
	Met (M)	9	4.3%
	Phe (F)	14	6.8%
	Pro (P)	10	4.8%
	Ser (S)	0	0.0%
	Thr (T)	18	8.7%
	Trp (W)	0	0.0%
	Tyr (Y)	16	7.7%
	Val (V)	13	6.3%
	Pyl (O)	0	0.0%
	Sec (U)	0	0.0%
Atomic composition	Carbon	C	1098
	Hydrogen	H	1709
	Nitrogen	N	247
	Oxygen	O	278
	Sulfur	S	9
Secondary structure analysis	Alpha helix (Hh) 53.14%<br>Extended strand (Ee) 25.12%<br>Random coil (Cc) 21.73%		
Solubility (SOLpro Server)	SOLUBLE with probability 0.994267		
