# Supplementary material for: Designing a Next-Generation Multiepitope-Based Vaccine against Staphylococcus aureus Using Reverse Vaccinology Approaches
Source: Pathogens. 2023 Feb 25;12(3):376. doi: 10.3390/pathogens12030376 (PMC10058999; doi:10.3390/pathogens12030376)
Supplement: Supplementary file 1 [file pathogens-12-00376-s001.zip › Supplementary Table S7.pdf]

Supplementary Table S7: Discontinuous B-cell epitopes with their scores predicted by ElliPro.

Sl. No.	Residue	Number of Residue	Score
A	A:A23, A:A24, A:K25, A:K26, A:D27, A:N28, A:Q29, A:D30, A:G31, A:K32	10	0.836
B	A:A108, A:A109, A:Y110, A:E111, A:F112, A:E113, A:V114, A:Q115, A:G116, A:R117, A:N118, A:L119, A:A120, A:A121, A:Y122, A:V123, A:K124, A:P125, A:T126, A:I127, A:Y128, A:F129, A:K130, A:L131	24	0.806
C	A:A133, A:Y134, A:N137, A:T138, A:V141, A:T142, A:A144, A:A145, A:Y146, A:I147, A:E148, A:Y149, A:T150, A:V151, A:T152, A:E153, A:D154, A:H155, A:A156, A:A157, A:L182, A:L183, A:I185, A:I186, A:T187, A:G188, A:P189, A:G190	28	0.718
D	A:M1, A:A2, A:I3, A:V4, A:G5, A:T6, A:K9, A:Y50, A:T51, A:P52, A:D53, A:G54, A:A55, A:T56, A:T58, A:F59, A:A60, A:A61, A:Y62, A:K63, A:V64, A:N65, A:G66, A:Y67, A:T68, A:T69, A:H70, A:V71, A:A72, A:A73, A:Y74, A:T75, A:T76, A:P77, A:E78, A:G79, A:Y80, A:T81, A:K82, A:K83	40	0.655
