# Supplementary figures and images for: Designing a Next-Generation Multiepitope-Based Vaccine against Staphylococcus aureus Using Reverse Vaccinology Approaches
Source: Pathogens. 2023 Feb 25;12(3):376. doi: 10.3390/pathogens12030376 (PMC10058999; doi:10.3390/pathogens12030376)

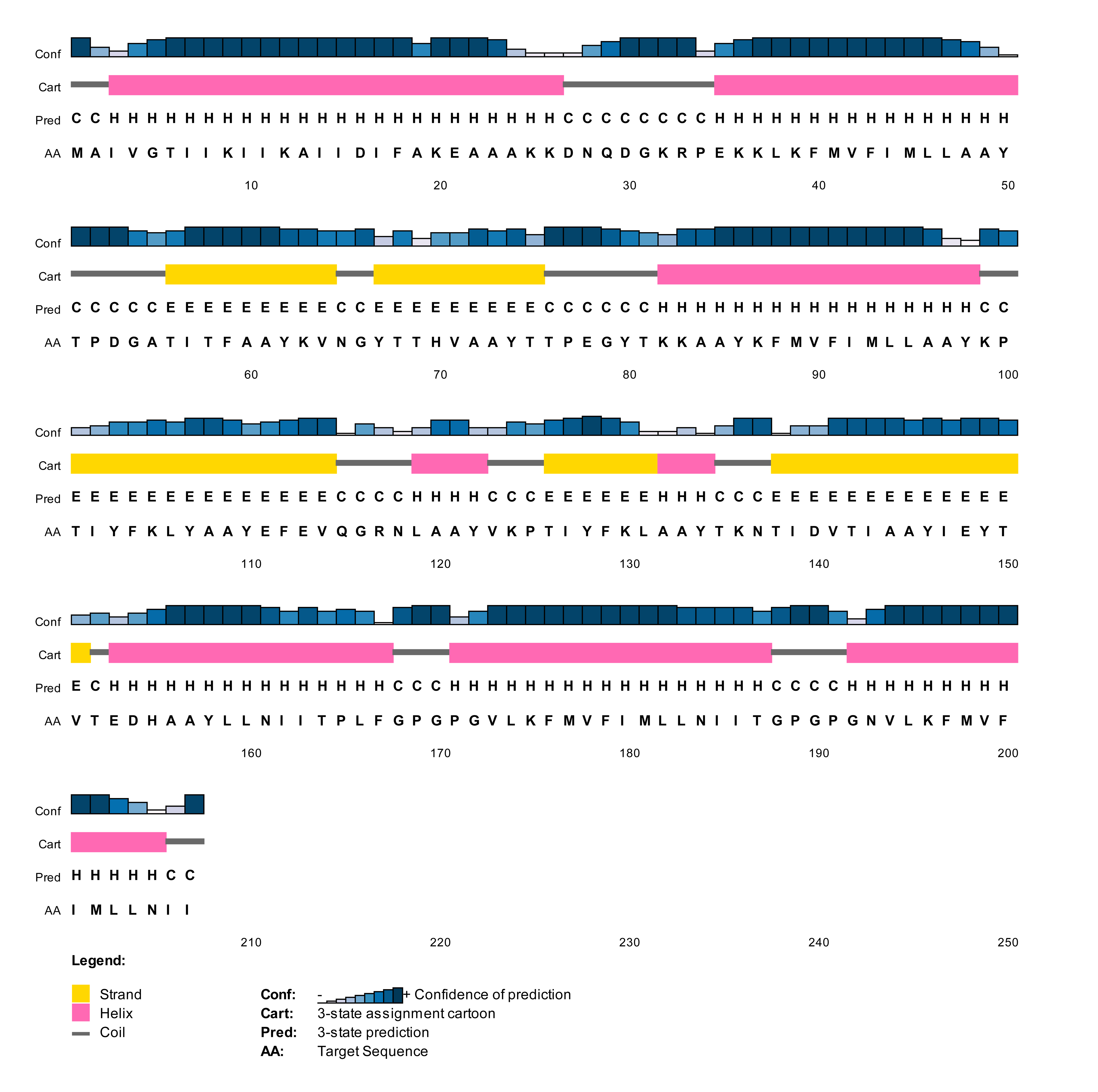

Supplement: Supplementary file 1 [file pathogens-12-00376-s001.zip › Supplementary Figure S1.jpg]

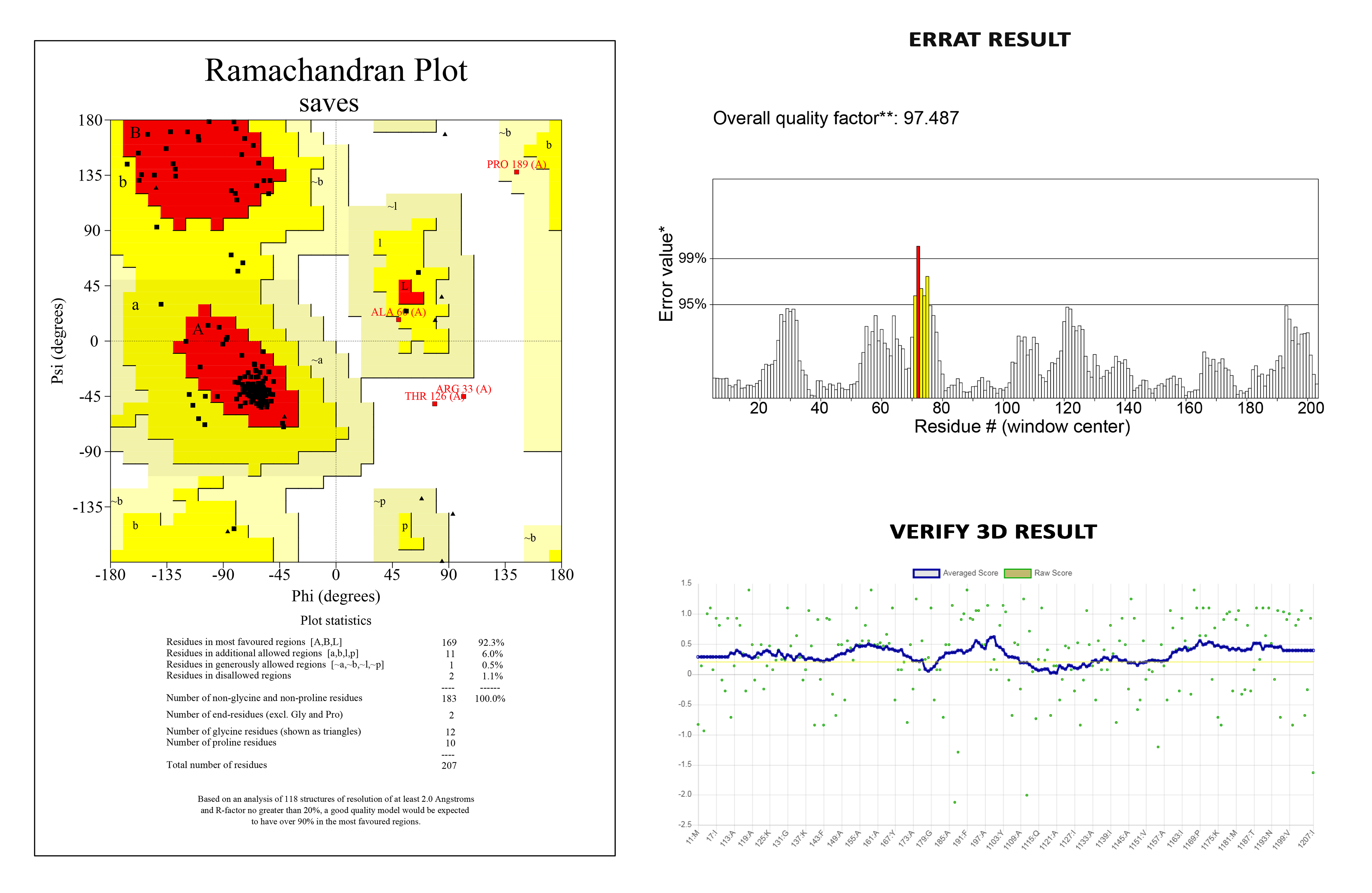

Supplement: Supplementary file 1 [file pathogens-12-00376-s001.zip › Supplementary Figure S2.jpg]
